# Neuroanatomical and neurochemical effects of prolonged social isolation in adult mice

**DOI:** 10.3389/fnana.2023.1190291

**Published:** 2023-08-17

**Authors:** Vibol Heng, Michael Zigmond, Richard Jay Smeyne

**Affiliations:** ^1^Department of Neuroscience, Thomas Jefferson University, Philadelphia, PA, United States; ^2^Department of Neurology, University of Pittsburgh, Pittsburgh, PA, United States

**Keywords:** isolation, enriched environment (EE), mouse, brain, hippocampus, cortex

## Abstract

**Introduction:**

As social animals, our health depends in part on interactions with other human beings. Yet millions suffer from chronic social isolation, including those in nursing/assisted living facilities, people experiencing chronic loneliness as well as those in enforced isolation within our criminal justice system. While many historical studies have examined the effects of early isolation on the brain, few have examined its effects when this condition begins in adulthood. Here, we developed a model of adult isolation using mice (C57BL/6J) born and raised in an enriched environment.

**Methods:**

From birth until 4 months of age C57BL/6J mice were raised in an enriched environment and then maintained in that environment or moved to social isolation for 1 or 3 months. We then examined neuronal structure and catecholamine and brain derived neurotrophic factor (BDNF) levels from different regions of the brain, comparing animals from social isolation to enriched environment controls.

**Results:**

We found significant changes in neuronal volume, dendritic length, neuronal complexity, and spine density that were dependent on brain region, sex, and duration of the isolation. Isolation also altered dopamine in the striatum and serotonin levels in the forebrain in a sex-dependent manner, and also reduced levels of BDNF in the motor cortex and hippocampus of male but not female mice.

**Conclusion:**

These studies show that isolation that begins in adulthood imparts a significant change on the homeostasis of brain structure and chemistry.

## Introduction

Humans are social animals, a characteristic that has been selected for during evolution (Buss, 1990). Given the importance of social interactions for humans, it is not surprising that social isolation leads to toxic physiological and psychological consequences (House et al., 1988; Cacioppo and Hawkley, 2003; Hawkley and Cacioppo, 2003; Weiss et al., 2004; Schiavone et al., 2013; Holt-Lunstad and Smith, 2016). Social isolation can be defined structurally as the absence of social interactions, contacts, and relationships with family and friends, with neighbors on an individual level, and with society at large on a broader level (Institute of Medicine (US) Division of Health Promotion and Disease Prevention et al., 1992). Isolation in humans can be effected by many conditions including, the estimated 3.3 million people currently housed in nursing homes or assisted living facility (Harris-Kojetin et al., 2019), 20% of the US population estimated to suffer from persistent loneliness (Wilson and Moulton, 2010), social isolation (distancing) due to a public health crisis, as in the case with the SARS-CoV2 virus in the U.S. (Abel and McQueen, 2020) as well as an extreme condition of isolation that occurs within the criminal justice system – that of incarceration in solitary confinement. A number of studies have examined the physiological and psychological effects of prolonged confinement in humans. Isolation has been shown to induce high rates of anxiety, panic, irritability, aggression, trouble sleeping, dizziness, perspiring hands, heart palpations, and increased prevalence of hypertension (Grassian, 2006; Haney, 2009, 2018). Psychologically, isolation induces hypersensitivity to external stimuli, hallucinations, panic attacks, cognitive deficits, obsessive thinking and paranoia, hopelessness, depression, social withdrawal, self-harm, and suicidal ideation and behavior (Smith, 2006; Cloyes et al., 2020; Reiter et al., 2020). Despite our understanding of the psychological and peripheral physiological effects of isolation, little is known of the anatomical or neurochemical changes that is induced by this isolation that begins in adulthood (Institute of Medicine (US) Committee on Ethical Considerations for Revisions to DHHS Regulations for Protection of Prisoners Involved in Research et al., 2007).

Despite the lack of studies on these structural and biochemical impacts of isolation in humans, some insight can be obtained from the extensive literature on early social isolation in rodents. Recent studies have demonstrated that rodents raised in isolation show structural changes in their brain, including reduced dendritic complexity and spine density in medial forebrain, nucleus accumbens (NAc) and CA1 hippocampus (Wang et al., 2012; Liu H. et al., 2020), as well as increased dendritic arborization in the basolateral amygdala (Wang et al., 2012). In addition, social isolation has been shown to impair myelination in the prefrontal cortex (PFC) (Kikusui et al., 2007; Liu et al., 2012; Makinodan et al., 2012), alter neurogenesis (Spritzer et al., 2011), decrease expression of synaptic proteins (Liu N. et al., 2020), alter hypothalamic pituitary adrenal (HPA) axis functioning (Weiss et al., 2004), and modify the levels of neurotrophins and monoamines in the brain (Heidbreder et al., 2000; Barrientos et al., 2003; Scaccianoce et al., 2006; Han et al., 2011; Berry et al., 2012; Garrido et al., 2013). Animals raised in isolation also manifest hyperactivity (Berry et al., 2012; Lander et al., 2017), impaired sensorimotor gating (Fone and Porkess, 2008; Wang et al., 2012), altered reversal learning (Han et al., 2011), neophobia, aggression, cognitive rigidity (Hall et al., 1997; Pinna et al., 2004; Fone and Porkess, 2008), increased anxiety- and depression-like behaviors (Westenbroek et al., 2003; Arakawa, 2005; Berry et al., 2012; Lander et al., 2017), and a reduced capacity to cope with stress (Friedler et al., 2015). Moreover, these animals also have increased morbidity and mortality (Cacioppo and Hawkley, 2003; Friedler et al., 2015; Holt-Lunstad and Smith, 2016; Razzoli et al., 2018). Some of these effects are sex-dependent and change with the age of animals and the duration of differential experience (Bartesaghi and Serrai, 2001; Bartesaghi and Severi, 2002; Pisu et al., 2016).

The issue with these studies, compared to our paradigm, is that they examine isolation from very early ages, such as those that examine effects of early maternal separation (Arling and Harlow, 1967; Helmeke et al., 2001; Kawano et al., 2008; Shams et al., 2012); whereas the vast majority of incidents of isolation in people occur in later adulthood and more than likely are seen in within the female population (Bruce et al., 2019). An exception to this is the isolation seen within the criminal justice system, which usually occurs in adulthood, but the majority are males (National Institute of Corrections, 2015). What is unique about this study is that we examine the effects of isolation in animals born and raised in an enriched environment and only then as adults, are transferred to isolated housing; again, this being the typical pattern for onset of isolation in humans. Here, mice were born in multigenerational enriched housing (Kempermann et al., 1997; Faherty et al., 2005). At the time of weaning (5 seeks of age) male and female mice were separated by sex but still maintained in enriched environment until they were 4 months of age. At this time, the animals were moved into an impoverished isolation. Within the isolated environments, animals were able to see, hear and smell other animals; i.e., we are examining the effects of impoverished isolation and not total sensory deprivation. After a period of 1 or 3 months of isolation; a time that corresponds to 3–5 human years (Flurkey et al., 2007), we measured its effects on several aspects of neuronal morphology, as well as catecholamine and neurotrophin levels. We found that isolation altered each of these variables in a time and sex-dependent manner.

## Materials and methods

### Animals

Male and female C57BL/6J mice (Jackson Laboratories, Bar Harbor, ME, USA) were used as the breeding stock for all animals used in this study. These animals were maintained in a temperature-controlled environment with *ad libitum* access to food and water in their home cages. Mice were kept in a temperature-controlled room under standard laboratory conditions, with a 12-h light/dark cycle (lights on at 6:00 a.m.) as well as constant temperature (22 ± 2°C) and humidity (55 ± 10%). Olfactory, visual, and auditory contact between the cages was not limited, whereas social interaction with the experimenter was limited to the handling during the weekly cage change. All animal procedures in this study followed the “NIH Guide for the Care and Use of Laboratory Animals” and were approved by the Institutional Animal Care and Use Committee of Thomas Jefferson University (TJU, protocol 00182).

### Experimental design and timeline

To generate animals in this study, 4 pregnant C57BL/6J female mice were placed into an enriched environment (Faherty et al., 2005) and allowed to give birth. All offspring were then co-raised by these 4 lactating dams. The enriched environment was a 1 m × 1 m polycarbonate enclosure that contained 3 running wheels, nesting materials (nestlets and cardboard huts), chewing toys (wood and balls) and a system of interchangeable tunnels that were re-arranged on a weekly basis. When the co-raised offspring of the original 4 pregnant dams reached 28 days of age, they were culled to cohorts of 14 male and 14 female mice and placed in a sex-segregated enriched environment until they were 4 months of age, which is considered to be young adulthood (Flurkey et al., 2007). To generate enough animals for this study, this was repeated 18 times. From each cohort of animals in the EE cages (progeny of 4 pregnant mice), half of the male and female mice in the enriched environment were then removed from the enriched environment and placed into social isolation consisting of single-housed standard cages measuring 7.25″ W × 11.5″ D × 5″ H. Once the mice were assigned and moved into these different environments, they were examined for changes at 1 and 3 months. For female mice, all analysis was not performed during estrous phase, based on the shape of the vaginal opening (Byers et al., 2012). Due to different methods used for tissue preparation, different cohorts of control and isolated mice animals were used for each analysis. generate animals for all of the studies, this process was repeated 16 times.

### Neuroanatomical analysis

#### Golgi cox staining

Animals were deeply anesthetized with Avertin, after which they were decapitated, and the fresh brains quickly removed from the calvaria and stained *en bloc* using the Rapid GolgiStain™ kit as directed by the manufacturer (FD Neurotechnologies Inc., Columbia, MD, USA). After *en bloc* staining, brains were rapidly frozen and serial 160-micron sections cut through from the forebrain to the midbrain/hindbrain junction (thus excluding the cerebellum, hindbrain, and spinal cord) and mounted on gelatin-coated microscope slides (Azer Scientific, Morgantown, PA, USA). Sections were allowed to dry naturally at room temperature in the dark for 1–3 days before being processed. Detection of neurons was performed using protocols in the Rapid GolgiStain™ kit. After staining, the sections were cleared in xylene 3 times for 4 min each and cover slipped with Permount^®^.

Images of Golgi-impregnated neurons were captured with Leica TCS SP8 confocal laser scanning microscope equipped with a HC PL APO CS2 40×/1.30 oil objective and the Perfect Focus System for maintenance of focus over time. The images were acquired in brightfield setting by using the 488 nm laser with Photomultiplier Tube (PMT) trans-On. In each image, z-series optical sections were collected with a step-size of 0.5 μm and an image stitching feature controlled by LASX software was used that captures all of the three-dimensional feature of the neuron of interest.

Reconstructed images of complete Golgi-impregnated neurons from sensory cortex, motor cortex and the CA1 region of the rostral hippocampus were analyzed using the Neurolucida 360 Program (MBF Biosciences, Williston, VT, USA). This program allows tracing of individually labeled Golgi-impregnated neurons, with the ability to trace different components of neurons (axon, dendrites, cell body, and spine) in the x, y, and z planes from captured z stacks, and record changes in process thickness. The neurons were chosen based on the following criteria: The neuronal body and dendrites were fully impregnated, there were less than 5% cut ends to the individual dendrites within the cell and the entire neuron was visible and relatively isolated from surrounding neurons. A total of 4–5 neurons/animal were examined and the mean of these values was determined; this mean value being the datapoint used in the analysis.

We analyzed neurons from layer II of sensory cortex (0.5 mm to 1.34 mm from the Bregma, plate 20–27), layer V of the motor cortex (0.5 mm to 1.34 mm from the Bregma, plate 20–27), and pyramidal neurons in the CA1 region of the hippocampus (−2.5 mm to −1.94 mm from the Bregma, plate 47–52) (61). Once each neuron was traced and the data recorded, four separate types of analysis were performed: total dendritic length per neuron, total volume (bounding box area), neuron complexity based on dendritic branching (Sholl analysis), and spine density (Lloyd et al., 2003).

### Biochemical analysis

For analysis of catecholamines and BDNF, male and female mice from each condition (Table 1) were deeply anesthetized with Avertin until reflexes are absent and then decapitated. Fresh brains were quickly removed from the calvaria and placed in a plastic brain mold cooled with dry ice. Razor blades were inserted at 2 mm intervals starting at midbrain/hindbrain junction. The 2 mm brain slices were laid out on cold mold plate and 4 brain regions: frontal cortex, motor cortex, striatum, and hippocampus were dissected and quickly frozen in dry ice and stored in −80°C.

**TABLE 1:** Number of animals used in each analysis.

Test	Male EE	Female EE	Male 1 m isolation	Female 1 m isolation	Male 3 m isolation	Female 3 m isolation
Golgi	16–24	11–17	29–30	19–20	29–40	30–40
Catecholamine analysis	15	10	10	5	5	6
BDNF	15	10	10	5	8	5

### Catecholamine analysis

We analyzed catecholamine content from striatum and frontal cortex from male and female mice in each condition (Table 1). Tissues were removed from −80°C storage and placed into dry ice prior to the addition of homogenization buffer to prevent degradation of biogenic amines. Tissues are then homogenized, using a handheld sonic tissue dismembrator, in 100–750 μl of 0.1 M TCA containing 0.01 M sodium acetate, 0.1 mM EDTA, and 10.5% methanol (pH 3.8). Ten microliters of homogenate from the sample were used for the protein assay. The samples were then spun in a microcentrifuge at 10,000 *g* for 20 min. Supernatant was removed for HPLC-ECD analysis. HPLC was performed using a Kinetix 2.6 μm C18 column (4.6 × 100 mm, Phenomenex, Torrance, CA USA). The same buffer used for tissue homogenization was used as the HPLC mobile phase. Concentrations were extrapolated from standards using a 5-point curve.

Protein concentration in tissue pellets was determined by Pierce BCA Protein Assay, cat 23227, ThermoFisher, Waltham, MA, USA. Ten microliter tissue homogenates were distributed into 96-well plate and 200 ml of mixed BCA reagent (25 ml of Protein Reagent A mixed with 500 μl of Protein Reagent B) was added. The plate was incubated at room temperature for 2 hrs for the color development. A BSA standard curve was run at the same time. Absorbance was measured by the plate reader (POLARstar Omega). DA turnover was estimated by determining the ratio of the sum of the major metabolites of DA synthesis [3,4-dihydroxyphenylacetic acid (DOPAC)] and homovanillic acid (HVA) divided by DA levels (DOPAC + HVA/DA).

### Determination of BDNF

Levels of the mature form of brain derived neurotropic factor (BDNF) were determined from frontal cortex and hippocampus of male and female in each condition (Table 1) using the BDNF Rapid ELISA Kit (Biosensis Pty Ltd., Thebarton, SA, Australia) according to manufacturer’s instructions. Briefly, brain tissues were re-suspended in an approximately 10 weight/volume ratio of acid-extraction buffer (50 mmol/L sodium acetate, 1 mol/L NaCl, 0.1% Triton x100, acetic acid, “Complete Mini” protease inhibitors cocktail tablet, pH 4.0). The suspension was then sonicated to homogeneity on ice with a Branson Digital Sonifier SFX150 in short bursts of 5 sec on and 5 sec off for the total of 30 s to avoid excessive sample heating. The homogenates were kept on ice for 30 min and another round of sonication as well as incubation on ice was performed. The homogenates were centrifuged for 30 min at 12,000 × *g* at 4°C. The clear supernatants were then transferred into clean tubes and total protein concentration measured using a DC protein assay (Bio-Rad, Hercules, CA, USA). To prepare for ELISA, sample dilution with 1 part tissue extracts and 3 parts of incubation/neutralization buffer (0.1 mol/L phosphate buffer, pH 7.6) were prepared. The final pH of sample was near neutral. ELISA assay was performed at room temperature. First, 100 μl of diluted mature BDNF standards, QC sample, samples and blank were added to the pre-coated microplate wells. The plate was then sealed with parafilm and incubated on a shaker (140 rpm) for 45 min. The solution inside the wells was then discarded and the wells washed 5 times with 1× wash buffer. After washing, 100 μl of the detection antibody was added into each well and the plate was sealed and incubated on a shaker for 30 min. After discarding the solution inside the wells and washing 5 times with 1× wash buffer, 100 μl of the 1× streptavidin-HRP conjugate was added into each well followed by 30 min of incubation on a shaker. The solution inside the wells was then discarded followed by 5 washes with 1× wash buffer. 100 μl of TMB was then added into each well and the plate incubated at room temperature for 6 min in the dark without shaking. The reaction was stopped by adding 100 μl of the stop solution into each well. Within 5 min after adding the stop solution, the absorbance was read at 450 nm with the Molecular Devices SpectraMax 384 Plus microplate reader. Results were reported as ng BDNF/mg total soluble protein.

### Statistical analysis

All the data are presented as mean ± SEM. Statistical analysis was performed using Prism 7 (GraphPad Software). The results of morphological changes for Golgi staining, biochemical changes for HPLC and BDNF ELISA were analyzed by three-way factorial MANOVA followed by simple effect analysis with Bonferroni adjustment if a single factor or interaction effect was statistically significant. Prior to analysis an outlier test at alpha = 0.01 was run on all data and values deemed outliers were removed from the analysis. A value of *p* < 0.05 was considered as statistical significance. Table 1 shows the number of cells or animals examined in each condition.

## Results

### Neuroanatomical effects of adult-induced isolation

Using both male and female adult mice, we examined the effects of two different periods of isolation (1 and 3 months) on total neuron volume, total dendritic length, dendritic complexity (branching), and spine density from three different regions of the CNS: Layer II neurons from the somatosensory cortex, Layer V neurons from the motor cortex, and CA1 pyramidal cells from the hippocampus.

### Effects of isolation Effects on neuronal volume

**FIGURE 1:**
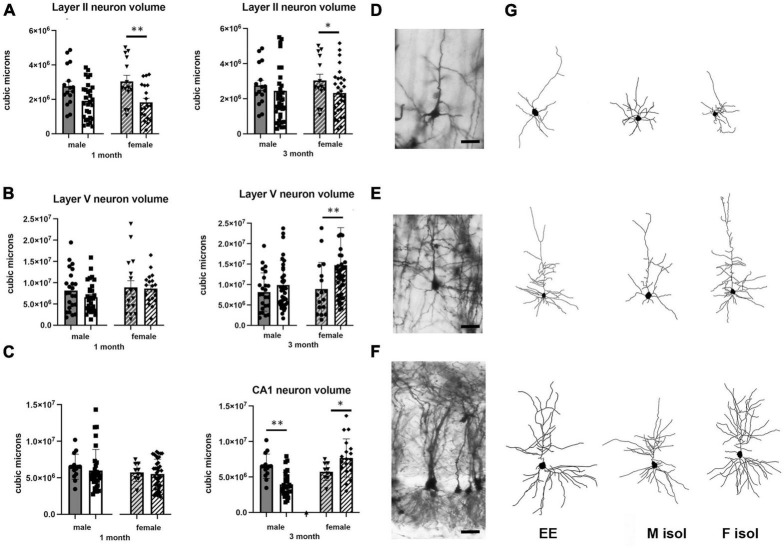
Effects of 1 and 3 months of isolation introduced as adults on volume of neurons located in **(A)** volume of neurons located in layer II from somatosensory cortex **(B)** volume of neurons located in layer V from motor cortex **(C)** volume of neurons located in Layer V of the motor cortex. **(D)** Appearance of Layer II neuron from somatorsensory cortex impregnated by Golgi method. **(E)** Appearance of Layer V neuron from motor cortex impregnated by Golgi method. **(F)** Appearance of CA1 neuron from rostral hippocampus impregnated by Golgi method. **(G)** Neurolucida 360 projection drawings of typical neurons from each regions examined from Enriched environment (EE), isolation from male mice and isolation from female mice. *p < 0.05, **p < 0.01. Scale bar **(A)** 40 μm, **(B,C)** 25 μm.

Neuronal volume, measured by placing a bounding box around the most distal ends of the axon and dendrites, was differently affected dependent on region of the brain examined as well as sex of the animal and duration of isolation. Bounding box volumes were compared from neurons in layer II neurons from the somatosensory cortex (Figures 1A, D), layer V neurons from the motor cortex (Figures 1B, E) and CA1 pyramidal cells from the hippocampus (Figures 1C, F).

In Layer II neurons of the somatosensory cortex, there was a statistically significant housing effect (*F*_1,170_ = 11.68, *p* < 0.0008), but no other sources of variation or interactions were measured. We found a significant reduction of neuronal volume by 40% at 1 month of isolation in female mice (*p* < 0.01) which lessened to 24% at 3 months of isolation compared to their EE housed littermates (*p* < 0.01). No significant differences in neuronal volume were seen in male in Layer II of the sensory cortex at either 1 or 3-months of isolation (Figures 1A, G).

In Layer V neurons of the motor cortex, there was a statistically significant effect of sex (*F*_1,203_ = 5.541, *p* < 0.0195) and time in isolation (*F*_1,203_ = 6.995, *p* < 0.0088) as well as an interaction housing and time in isolation (*F*_1,203_ = 6.995, *p* < 0.0088). Specifically, we found a significant 67% increase of neuronal volume in female mice at 3 months of isolation compared to their EE littermates (*p* < 0.001) (Figures 1B, G).

In the CA1 pyramidal neurons in the hippocampus, there were significant two-way interactions between sex and time in isolation (*F*_1,157_ = 7.946, *p* < 0.0054) and sex and housing (*F*_1,157_ = 10.44, *p* < 0.0054). At 1 month of isolation, no significant differences in neuronal volume were seen in CA1 pyramidal neurons of male or female mice. However, after 3 months of isolation male mice showed a significant 39% decrease in the volume of CA1 neurons (*p* < 0.001) while female mice exhibited a significant 34% increase compared to EE housed littermates (*p* < 0.02) (Figures 1C, G).

### Effects of isolation effects on dendrite morphology

**FIGURE 2:**
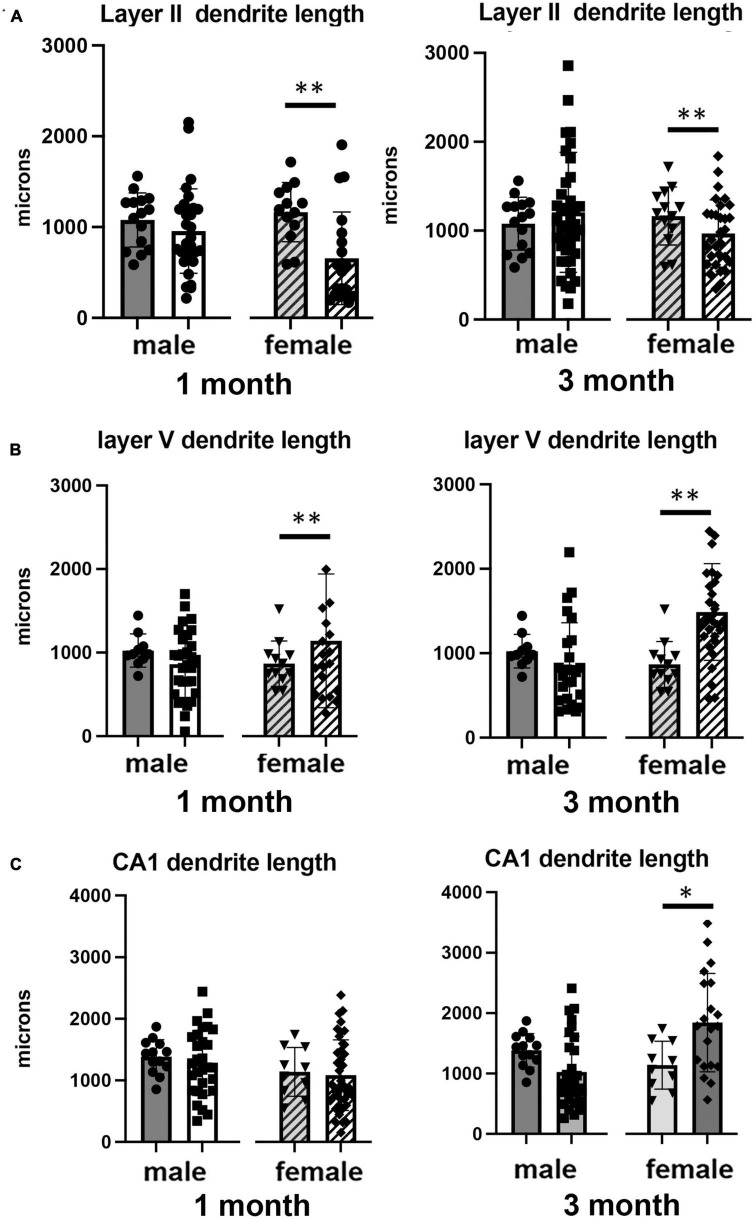
Effects of 1 and 3 months of isolation introduced as adults on total dendrite length from **(A)** neurons in layer II from somatosensory cortex, **(B)** layer V from motor cortex and **(C)** CA1 neurons from the rostral hippocampus of male and female mice. *p < 0.05, ^**^p < 0.01.

**FIGURE 3:**
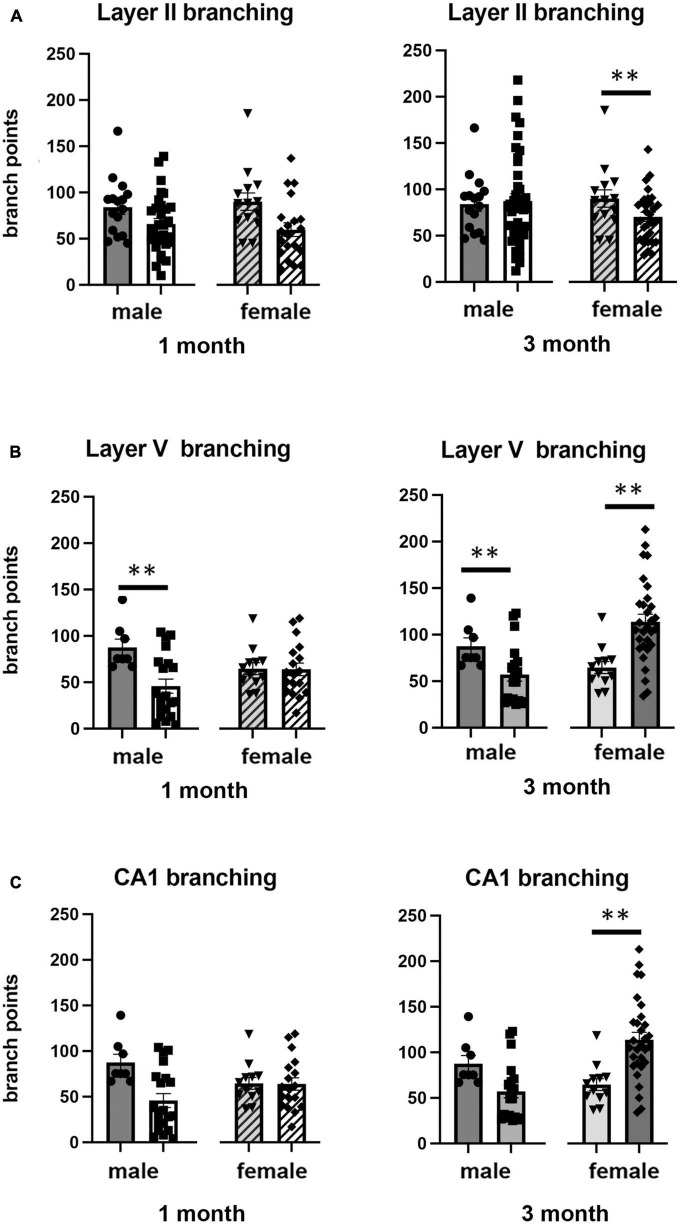
Effects of 1 and 3 months of isolation introduced as adults on neuronal complexity (dendritic branching) from **(A)** neurons in layer II from somatosensory cortex, **(B)** layer V from motor cortex and **(C)** CA1 neurons from the rostral hippocampus of male and female mice. ^**^p < 0.01.

**FIGURE 4:**
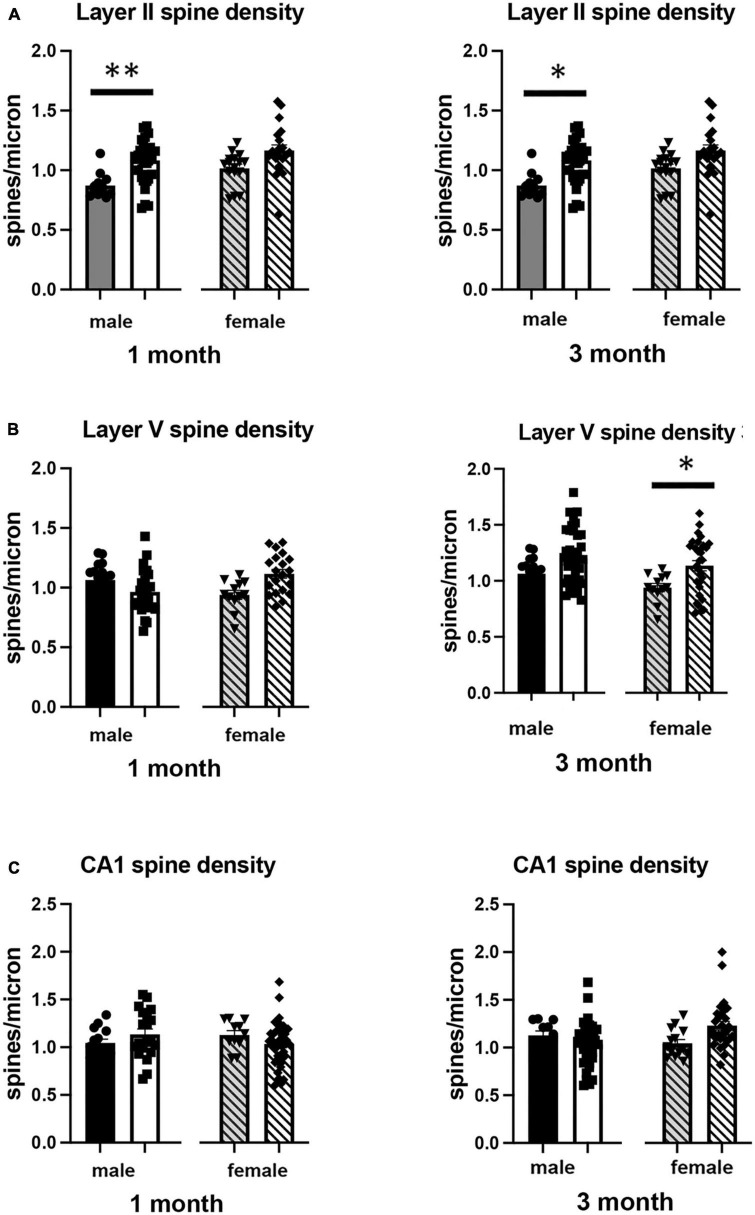
Effects of 1 and 3 months of isolation introduced as adults on spine density from neurons in **(A)** layer II from somatosensory cortex, **(B)** layer V from motor cortex and **(C)** CA1 neurons from the rostral hippocampus of male and female mice. *p < 0.05, ^**^p < 0.01.

We also examined the effect of 1 or 3 months of isolation in male and female adult mice on three aspects of dendrite morphology: the total length (Figure 2), complexity (branching) (Figure 3), and spine density (Figure 4) from neurons in Layer II of the somatosensory cortex, Layer V of the motor cortex and CA1 hippocampal pyramidal cells.

In Layer II of the somatosensory cortex, there was a significant two-way interaction between sex and housing condition on total processes length (*F*_1,168_ = 5.190, *p* = 0.026) and a significant main effect of isolation on dendritic branching (*F*_1,172_ = 7.711, *p* < 0.006) and dendritic spine density (*F*_1,168_ = 22.49, *p* < 0.0001). Examination of housing effects shows a significant reduction of total processes length by 44% in female mice at 1 month (*p* < 0.007, Figure 2A), with no changes seen in male mice. Female mice also show a significant 30% decrease in dendrite branching at 3 months (*p* < 0.007). In regard to spine density, there was a significant 21% increase in dendritic spine density in male mice (*p* < 0.009) that remains elevated at 3 months (*p* < 0.03) (Figure 4A).

In Layer V of the motor cortex, there was a significant two-way interaction between sex and housing condition on total neurite processes length, dendritic branching, and dendritic spine density (*F*_1,141_ = 11.60, *p* < 0.0009; *F*_1,120_ = 22.02, *p* < 0.0001; *F*_1,163_ = 6.34, *p* = 0.013, respectively).

Examination of housing effects shows no change in dendrite length at 1 month in male mice. In female mice, we did measure a 31% increase in female mice (*p* < 0.01). At 3 months, this increase in dendrite length increased to 71% increase in female mice (*p* < 0.01, Figure 2B). As with the 1 month timepoint, no changes were observed after 3 months in male mice. Significant changes were also measured in the number of dendritic branches in both male and female mice. Male mice demonstrated a 47% decrease in dendrite branching at 1 months (*p* < 0.01) that remains reduced at 3 months. Female mice also show significant changes in dendrite branching, but unlike males, after 3 months of isolation, we observed a 75% increase in branches (*p* < 0009) (Figure 3B). In regard to spine density, we only observed significant changes in female mice after 3 months of isolation, where there was a significant 21% increase in dendritic spine density (*p* < 0.02) (Figure 4B).

In Layer the CA1 region of the rostral hippocampus there was a significant two-way interaction between sex and housing condition on total neurite processes length, dendritic branching, and dendritic spine density (*F*_1,148_ = 6.659, *p* < 0.03; *F*_1,159_ = 9.475, *p* < 0.0003; *F*_1,148_ = 11.89, *p* = 0.0007, respectively). Examination of housing effects shows no change in dendrite length at 1 or 3 months in male mice and 1 month in female mice. However, we did measure a significant 62% increase in total processes length in female mice after 3 months of isolation (*p* < 0.02, Figure 2C). A similar pattern was seen in dendritic branching, where no change was seen in male or female mice at 1 month. We did measure a 69% increase in the number of dendritic branches in female mice after 3 months of isolation (*p* < 0009) (Figure 3C). In regard to spine density, no significant changes were seen after either 1 or 3 months of isolation in male and female mice (Figure 4C).

### Effects of isolation on CNS neurochemistry

**FIGURE 5:**
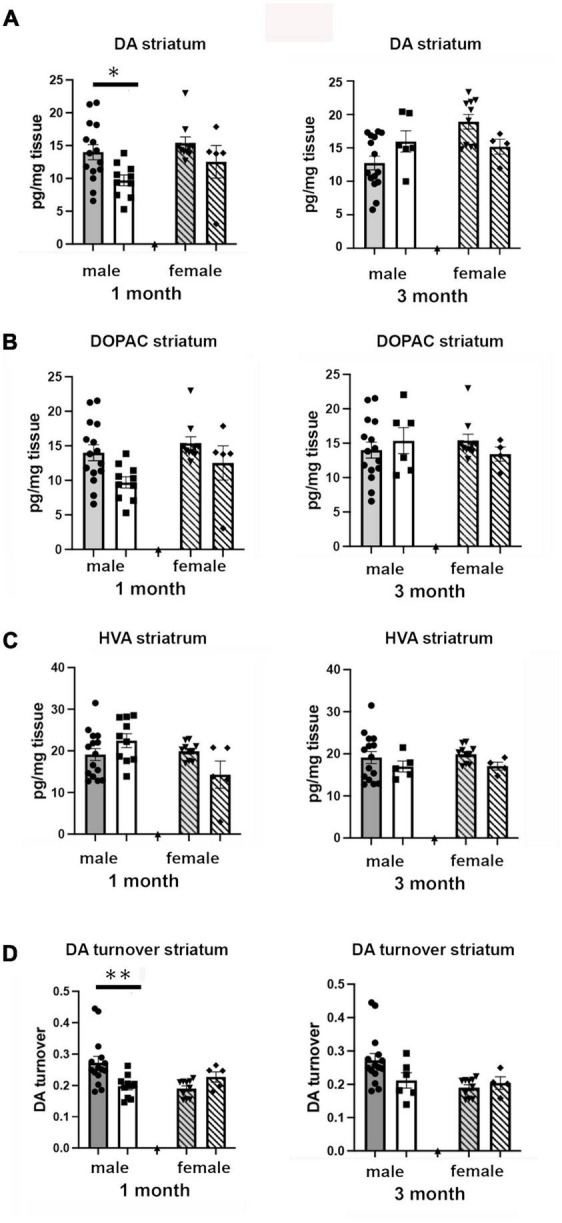
Effects of 1 and 3 months of isolation introduced as adults on **(A)** striatal dopamine, **(B)** DOPAC, **(C)** HVA and **(D)** DA turnover (DOPAC + HVA/DA) of male and female mice. *p < 0.05, ^**^p < 0.01.

We examined the effects of 1 and 3 months of isolation in adult male and female mice on levels of catecholamines in the striatum. In the striatum, levels of norepinephrine (NE) and serotonin (5-hydroxytryptamine, 5-HT) were only measured at or below the level of detection; both in enriched environment and isolation conditions (data not shown). Thus, any changes in these two neurotransmitters could not compared between conditions. We were able to detect dopamine (DA), 3,4-Dihydroxyphenylacetic acid (DOPAC), and homovanillic acid (HVA). We found that there were significant two-ways interaction between sex and housing on the level of DA, and DA turnover (estimated as the DOPAC + HVA/DA ratio) in the striatum (*F*_1,67_ = 16.72, *p* = 0.0001; *F*_1,67_ = 10.05, *p* < 0.002). In female mice, no significant difference in the levels of DA, DOPAC, HVA, and DA turnover was observed in the striatum after 1 or 3 months of isolation (Figures 5A–D). In male mice, 1 month of isolation induced a 32% increase in the level of striatal DA (*p* < 0.03), which normalized after 3 months of isolation (Figure 5A). The increase in the level of DA in the striatum in male mice at 1 month of isolation occurred together with a significant decrease in DA turnover, which was reduced by 43% after 1 month of isolation (*p* < 0.0005) (Figure 5D). No significant difference in DA turnover was observed in male mice after 3 months of isolation (Figure 5D).

Examination of catecholamine levels in the frontal cortex showed significant two-way sex x housing interactions in levels of norepinephrine (*F*_1,67_ = 14.86, *p* < 0.0023) but not in levels of DA or 5-HT. In regard to NE, no changes were detected in male mice after 1 or 3 months of isolation. However, in female mice, no change was detected after 1 month, but after 3 months isolation the levels of NE decreased by 54% (14.66 pg/mg tissue in EE versus 6.74 pg/mg tissue; *p* < 0.0001).

### Effects of isolation on hippocampal BDNF

**FIGURE 6:**
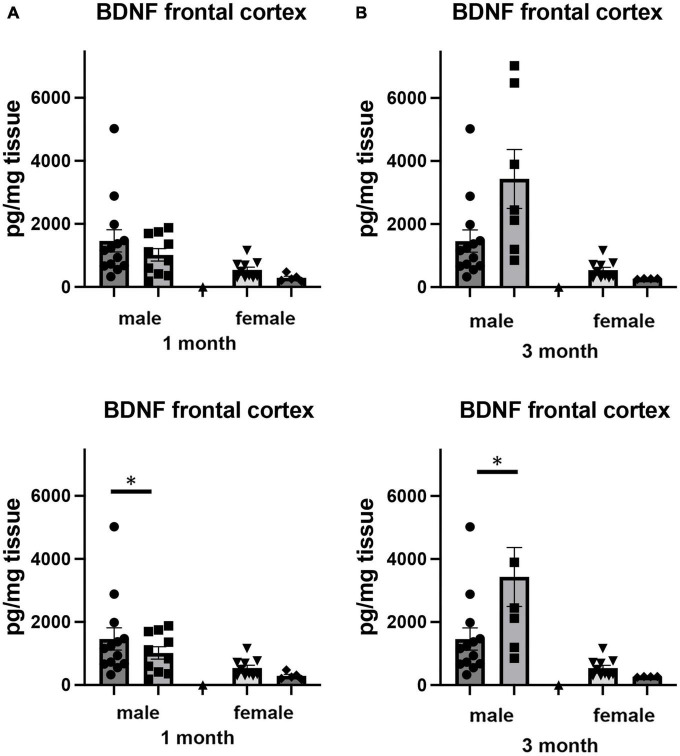
Effects of 1 and 3 months of isolation introduced as adults on BDNF levels in **(A)** cortex and **(B)** rostral hippocampus of male and female mice. *p < 0.05.

Brain derived neurotrophic factor, a member of the neurotrophin family of growth factors, has been shown to play critical roles in a number of cellular processes, including cell survival and differentiation (Snider and Johnson, 1989; Reichardt, 2006). In the brain, the level of BDNF has been shown to be labile, altering its levels in response to any number of cellular stresses, including those associated with social isolation (Murínová et al., 2017). We examined how adult-induced isolation altered the level of BDNF in frontal cortex and hippocampus (Figure 6). We found two-way sex x housing interactions when comparing BDNF in frontal cortex (*F*_1,64_ = 4.517, *p* < 0.037) as well as in hippocampus (*F*_1,68_ = 5.448, *p* < 0.023). In female mice, there was no significant difference in the level of BDNF in the frontal cortex at 1 month of isolation. However, after 3 months, we measured a 51% decrease (*p* < 0.05) (Figure 6A). In male mice, no significant changes were detected after 1 or 3 months from frontal cortex.

In hippocampus, we there were significant two-way interactions between sex and housing for BDNF (*F*_1,68_ = 5.448, *p* = 0.022). In female mice, there was no significant difference in the level of BDNF in the hippocampus at 1 or 3 months of isolation (Figure 6B). In male mice, 1 month of isolation induced 64% decrease in the level of BDNF (*p* < 0.05). After 3 months of isolation, the level of BDNF remained 31% lower than in mice in the EE conditions (Figure 6B).

## Discussion

In this study we examined the effects of 2 periods of isolation imposed on adult male and female C57BL/6J mice that had been born and raised in an enriched environment. We found that mice raised in an EE and subsequently isolated at 4 month of age had significant alterations in their neuronal morphology including changes in neuronal volume, dendritic length and branching, and spine density. These changes were dependent on brain region and period of isolation, as well as sex of the animal.

There is a significant literature on the neuroanatomical and neurochemical effects of housing. The vast majority of these studies examine the effects of environmental enrichment by comparing where animals housed in standard “shoebox” cages, either in small groups or individually, and then placed as adults in enhanced housing conditions with additional social interactions, availability of toys, exercise, and mentally stimulating apparatus (Honess and Marin, 2006; Gelfo et al., 2009; Segovia et al., 2009; Ashokan et al., 2016; Kempermann, 2019; Ohline and Abraham, 2019). However, whereas these studies can provide clues to potential plasticity of the brain, the temporal order of these modifications is far from that experienced by humans, where–except for the few instances of early life isolation (Sheridan et al., 2012)–we are born and raised in relative enrichment and then in later life are by circumstance isolated. These include isolating circumstances such as placement into nursing homes and other rehabilitation facilities, segregation from society into prisons and further into solitary confinement, and even by separation from general society by restrictions imposed by a viral pandemic. Additionally, despite the body of literature available about enrichment, one cannot just assume that the effects of isolation would be the opposite of enrichment, since moving from isolation to enriched environment does not necessarily equal that of moving from enriched environment to isolation. Thus, it is critical to examine these parameters in a temporally relevant way.

It has been shown that isolation during the early stages of development, particularly pre-weaning (i.e., maternal deprivation) and immediately after weaning, can have significant effects on brain structure (Wang et al., 2012; Maccari et al., 2014; Nishi, 2020). In fact, it appears that this form of deprivation is negatively correlated to the age when it is first introduced (Lapiz et al., 2003), with earlier isolation resulting in much more significant effects. However, this conclusion is based on only a very limited number of studies that have assessed the impact of social isolation introduced as adults after the pre/peri-weaning period. In regard to neuronal morphology, Liu N. et al. (2020) demonstrated that both 6–8-weeks-old male and female C57BL6/J mice isolated for 8 weeks had significantly decreased CA1 apical and basal branch points and reduced dendritic length and spine density compared to group-housed mice. Similar changes in neuronal structure were found in 45-day isolated middle aged rats (450 days), where it was found that isolation (compared to enriched environment) effected shorter terminating branches and less second and fifth order branches in both layer IV stellate and layer III pyramidal cells in the occipital cortical (Green et al., 1983). Additionally, the isolation induced in these studies followed separation from other rodents from group housing (3–4 animals/shoebox cage) that was otherwise devoid of enrichment. An additional confound to these studies was that Liu et al. examined these morphological changes after the mice were used for behavioral testing, i.e., they were handled prior to the anatomical analysis rather than being examined in sentinel animals.

In terms of adult-imposed isolation effects on brain neurochemistry, our study looked at baseline changes induced by isolation, whereas it appears that the few studies that have examined the effects of adult isolation studied these in context of response to exogenous stressors (Garrido et al., 2013). We found that the baseline levels of catecholamines and their metabolites to determine their turnover altered by time in isolation were dependent on the region examined, the time in isolation, and the sex of the animal. Male mice demonstrated a significant alteration in the striatal DA system, whereas isolation had no effect on the DA systems of female mice. Isolated female mice, on the other hand, had a significant alteration in the NE system in the frontal cortex, which was not affected in isolated male mice. The differential change in baseline neurochemistry (Konradi et al., 1992; Bangasser et al., 2016) (i.e., the “intrinsic reserve” (Stern et al., 2020) could be one reason we observed sex-dependent changes in isolation-induced behaviors. Our results are consistent with those observed following 8–9 weeks of isolation in male post-weaning rats, where social isolation led to no change in DA levels in mPFC compared to group-reared controls (Wang et al., 2012), no effect on basal extracellular 5-HT levels (Hall et al., 1998), and increased basal extracellular DA in the striatum measured by HPLC (Holson et al., 1991). In addition, our results with isolated female mice are consistent with those observed following prolonged isolation in female post-weaning rats, where social isolation led to decreased DA turnover in the PFC (Powell et al., 2003).

The vast majority of research on the expression of the neurotrophin BDNF has examined either peri- and early post-weaning animals (Murínová et al., 2017; Zaletel et al., 2017) or how these growth factors change in response to enriched environment as an adult (Torasdotter et al., 1998; Ickes et al., 2000; Faherty et al., 2005; Bekinschtein et al., 2011). In our studies, only isolated adult male mice showed significant reductions in the concentration of BDNF in the hippocampus, whereas female mice did not show any significant change in this regions. Our BDNF findings are also similar to several previous studies that found adult male mice and rats isolated from 3 to 14 weeks had decreased BDNF expression in the hippocampus (Scaccianoce et al., 2006; Berry et al., 2012; Liu N. et al., 2020). In addition, studies of the effects of isolation on BDNF expression have found that the neurotrophin was decreased (Scaccianoce et al., 2006), increased (Meng et al., 2011), or unchanged (Weintraub et al., 2010) in the PFC and hippocampus. This discrepancy may be indicative of sex, age, and strain differences in neurochemical alteration following isolation or may be influenced by the different isolation duration and molecular paradigms used in their study.

So how do these studies relate to and impact conditions of isolation in humans? Due to the longitudinal nature of isolation, the cost issues of access to non-invasive tools of measurement (e.g., CT scan and MRI), and in the case of persons isolated in the criminal justice system the capacity to obtain truly informed consent, only a few studies have examined isolation on brain size. One study examined brain size in eight male and female polar expeditioners who spent 14 months isolated in Antarctica at the German Neumayer III station. They found that there was a 7.2% loss of volume in the dentate gyrus of the hippocampus as well as smaller but significant changes in other regions of the brain including the PFC (Stahn et al., 2019). This study also examined serum BDNF levels and found that this was reduced by 45% (Stahn et al., 2019), similar to the reductions we measured in regions of brains of male mice. Other forms of isolation have measured similar shrinkage in the amygdala (Düzel et al., 2019). Due to similar factors described above, examination of catecholamines and growth factors in living brains of isolated people has not been reported. In fact, it is for these reasons that studies more closely representing isolating conditions in humans must be carried out using animal models. Although not directly measured in this study, there is also an excellent correlation between the behavioral manifestations of isolation experienced by people in isolation and those observed in mice, including increased anxiety, aggression, and depression (Matsumoto et al., 2005; Brenes and Fornaguera, 2009; Teo et al., 2013; Mumtaz et al., 2018; Takahashi et al., 2018; Reiter et al., 2020); thus supporting the use of animal modeling to predict the human experience.

Of all of the conditions of isolation experienced by humans, the model we use in this study best replicates that of the condition of solitary confinement that is used in the criminal justice system. In our studies as well as incarceration in solitary confinement, the isolation starts after a period of relative enrichment. Our model also reliates this human condition since we impose isolation at the approximately the age when a majority of the cases of solitary confinement occurs, which is equivalent to that of a young adult (Beck, 2015). Solitary confinement – for even short periods of time (days to weeks)–has been shown to induce a number of psychiatric disorders including hypersensitivity to external stimuli, hallucinations, panic attacks, cognitive deficits, obsessive thinking, and paranoia. Prolonged confinement also leads to numerous other negative symptoms, including loss of emotional control, mood swings, hopelessness, and depression, social withdrawal, and self-harm and suicidal ideation and behavior (Grassian, 1983, 2006; Haney, 2006, 2009; Reiter et al., 2020). In addition, persons who have experienced long-term solitary confinement may show memory loss and impaired concentration, and may report feeling extremely confused and disoriented in time and space (Scharff, 2006). However, no studies have been reported that examined any neuroanatomical or neurochemical changes in persons within this population.

One of the more surprising findings in this study is that social isolation started in adulthood appears to differently affect male and female animals. The differential impact of social isolation between human males and females has been well documented; and this is the major reason we chose to specifically examine male and female mice as individual cohorts. Some examples of differences in response to isolation include the finding that females have a significantly higher stress response than males (Senst et al., 2016; Liu H. et al., 2020), that females experience greater levels of depression and anxiety after isolation (Martin and Brown, 2010; Warner et al., 2019), and that females experience greater feelings of separation than males (Aranda-Hughes et al., 2021). A number of studies have shown that HPA signaling is different in male vs. female animals experiencing isolation (Cacioppo et al., 2011; Pisu et al., 2016; Mumtaz et al., 2018). Our studies show differences in catecholamine levels and neurotrophin levels between sexes. One unexpected observation was that in some of our neuronal analyses (dendrite length and branching) and we found that males and females had effects in opposite directions, rather than just greater or lesser responses in a single direction. At this time, we do not understand this difference; however, we hypothesize that any change from that measured from animals in continued exposure to an enriched environment would disrupt functional homeostasis; and thus have negative consequences. Still to be determined is whether any of the changes we measured are reversible, and if not, whether there a critical amount of time in isolation after which changes are permanent.

In conclusion, our studies in mice show that a relatively short period of isolation started in adulthood can induce significant changes in neuron size, dendrite length and spine density (which will affect its connectivity (Chklovskii, 2004), and changes in its intrinsic biochemistry, each likely to manifest alterations in the animal’s cognitive functioning.

## Conclusion

Overall, this body of work fills several gaps in the literature on social isolation by focusing on how different periods isolation in male and female mice, enforced as adults after being raised in relative enrichment affect neuron structure and biochemistry. These findings have substantial value in identifying both neurobiological and behavioral disturbances that occur secondary to isolation and thus, may be used to inform the development of therapeutic interventions in adults. Additionally, understanding how isolation changes the brain may provide a mechanism for predicting which disturbances in behaviors, as well as mental health, may occur in response to prolonged isolation. This may allow psychologists, clinicians, and community health leaders to employ evidence-based prevention programs to mitigate the risk of isolation-induced mental illness.

## Data availability statement

The original contributions presented in this study are included in this article/supplementary material, further inquiries can be directed to the corresponding authors.

## Ethics statement

The animal study was approved by Thomas Jefferson University IACUC. The study was conducted in accordance with the local legislation and institutional requirements.

## Author contributions

VH, MZ, and RS contributed to the conception and design of the study. VH and RS performed the experiments. VH wrote the first draft and the manuscript. All authors contributed to the manuscript revision and read and approved the submitted version.
